# Bioartificial Livers Developed From Gene‐Edited Pig Hepatocyte Organoids Improve Amino Acid and Lipid Profiles in the Plasma of Patients With Liver Failure

**DOI:** 10.1002/mco2.70795

**Published:** 2026-05-31

**Authors:** Yuting He, Yang Deng, Xinglong Zhu, Mengyu Gao, Qin Liu, Wanliu Peng, Yanyan Zhou, Lang Bai, Ji Bao

**Affiliations:** ^1^ Department of Pathology Institute of Clinical Pathology Key Laboratory of Transplant Engineering and Immunology NHC West China Hospital Sichuan University Chengdu China; ^2^ Department of Pathology West China Second University Hospital Sichuan University Chengdu China; ^3^ Key Laboratory of Birth Defects and Related Diseases of Women and Children (Sichuan University) Ministry of Education Chengdu China; ^4^ Department of Medical Genetics/Prenatal Diagnostic Center West China Second University Hospital Sichuan University Chengdu China; ^5^ Center of Infectious Diseases West China Hospital Sichuan University Chengdu China

**Keywords:** bioartificial liver, gene editing, liver failure, liver organoids, metabolomics

## Abstract

Liver failure remains a life‐threatening syndrome where the available therapeutic options are extremely limited beyond transplantation. This study addresses critical cell source and mechanistic challenges by developing a novel bioartificial liver (BAL) system. We utilized CRISPR/Cas9 technology to knockout the *GGTA1* gene in primary porcine hepatocytes to reduce immunogenicity. These hepatocytes were co‐cultured with R‐spondin1‐overexpressing human umbilical vein endothelial cells (R‐HUVECs) to form functionally stable liver organoids. In ex vivo study using plasma from patients with acute‐on‐chronic liver failure (ACLF), the BAL system demonstrated superior detoxification, significantly reducing ammonia and bilirubin levels compared to traditional non‐bioartificial liver (NAL) support. Multi‐omics analyses revealed that BAL treatment actively restored metabolic homeostasis by promoting branched‐chain amino acid (BCAA) metabolism and upregulating lysophosphatidylcholine (LPC) species associated with membrane repair and anti‐inflammatory signaling. Significantly, this research demonstrates that unlike the passive physical filtration of NAL, BAL serves as an active biological regulator of systemic metabolism. These findings provide a robust theoretical and practical foundation for the clinical translation of BAL technology, offering a promising strategy to improve outcomes for liver failure patients by modulating systemic metabolism.

## Graphical abstract

A bioartificial liver (BAL) integrating GGTA1‐knockout porcine hepatocytes and R‐spondin1‐overexpressing endothelial organoids was tested using plasma from patients with acute‐on‐chronic liver failure (ACLF). The BAL system actively modulates amino acid and lipid metabolism, enhances detoxification, partially restores overall metabolic balance, and outperforms conventional non‐bioartificial liver therapy, offering a promising approach for future liver support.

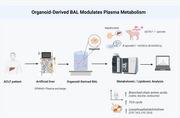

## 1 Introduction

Globally, liver‐related diseases account for approximately 1 million deaths annually. Liver failure represents a critical clinical condition characterized by a rapid decline in hepatic function, leading to metabolic collapse and multi‐organ dysfunction. A significant proportion of this mortality is attributed to acute liver failure (ALF) and acute‐on‐chronic liver failure (ACLF); the latter is defined as a distinct, inflammation‐driven syndrome associated with high short‐term mortality [1, 2]. Although liver transplantation remains the definitive treatment for end‐stage disease [3, 4], its clinical application is severely constrained by persistent challenges, including a chronic shortage of donor organs, prohibitive medical expenditures, and inherent surgical risks [3, 5].

Artificial liver (AL) support systems have been developed as bridging therapies for liver failure. Non‐bioartificial liver (NAL) systems, such as the double plasma molecular adsorption system (DPMAS), facilitate the rapid clearance of toxins through physical methods to delay disease progression. However, these systems lack the metabolic and synthetic functions of the native liver, which constrains their therapeutic efficacy. In contrast, bioartificial liver (BAL) systems utilize living hepatocytes to provide essential biological functions and are considered an alternative therapeutic approach [5, 6]. Survival benefits and neuroprotective effects of BAL systems have been demonstrated in non‐human primate and porcine models of ALF [7]. Furthermore, the capacity of BAL systems to enhance survival and promote liver regeneration has been reported in several studies [8, 9, 10].

The clinical translation of BAL technology is limited by several factors. First, safety concerns regarding the immunogenicity of xenogeneic cells and the risk of viral transmission, such as porcine endogenous retrovirus (PERV), restrict widespread clinical application [10, 11, 12]. Second, for various cell sources—including the primary porcine hepatocytes, transdifferentiated cells, and GMP‐grade induced hepatocytes—the maintenance of long‐term cellular functionality in vitro and the implementation of large‐scale cultures remain technical challenges [7, 13, 14]. Finally, although the efficacy of BAL systems has been demonstrated, the underlying molecular mechanisms, particularly the metabolic regulation of pathological states, have not been systematically elucidated [15, 16].

This study was designed to address these fundamental challenges. Diverging from the existing literature, this work circumvents the reliance on complex cell reprogramming or immortalization protocols; instead, primary porcine hepatocytes were utilized, and their primary limitations were specifically addressed. CRISPR/Cas9 gene‐editing technology was employed to knock out the GGTA1 gene, thereby diminishing the immunogenicity of porcine hepatocytes and providing a safer, more scalable source of primary cells [17, 18]. Concurrently, functionally stable liver organoids were established through the co‐culture of these hepatocytes with human umbilical vein endothelial cells (R‐HUVECs) overexpressing the R‐spondin1 (RSPO1) protein.

The high expression of RSPO1 is of paramount importance as it acts as a potent ligand to activate the Wnt/β‐catenin signaling pathway, which is a key driver of hepatocyte proliferation and regeneration. By providing a sustained R‐spondin1‐rich niche, this co‐culture system effectively overcomes the traditional bottleneck of primary cells, ensuring their long‐term survival and functional maintenance in vitro [19, 20, 21, 22]. Compared to existing BAL studies using primary porcine hepatocytes, which predominantly focus on enhancing cell functionality through the optimization of 3D scaffolds or bioreactor oxygenation, our approach offers a distinct paradigm by synergizing genetic xeno‐neutralization with proactive signaling niche reconstruction. While traditional primary cell‐based systems often struggle with the inherent immunogenicity of wild‐type porcine cells and the rapid loss of hepatocyte‐specific markers in vitro, the integration of *GGTA1*‐KO cells with a vascular‐mimetic RSPO1‐secreting environment ensures both immunological safety and sustained biological potency [23, 24, 25, 26]. This strategy avoids the complexities of cell reprogramming while significantly surpassing the functional longevity of standard primary porcine hepatocyte cultures. Furthermore, through the implementation of preclinical investigations utilizing plasma from patients with acute‐on‐chronic liver failure (ACLF) and the execution of comprehensive metabolomic analyses, the distinct metabolic regulatory mechanisms of the BAL system were systematically elucidated. This study effectively addresses a prominent lacuna in the current literature, thereby furnishing a more substantial theoretical framework for its clinical translation [27, 28, 29].

## 2 Results

### 2.1 Oviductal Injection of rAAV‐CRISPR/Cas9 for the Production of Gene‐Edited Pigs

**FIGURE 1:**
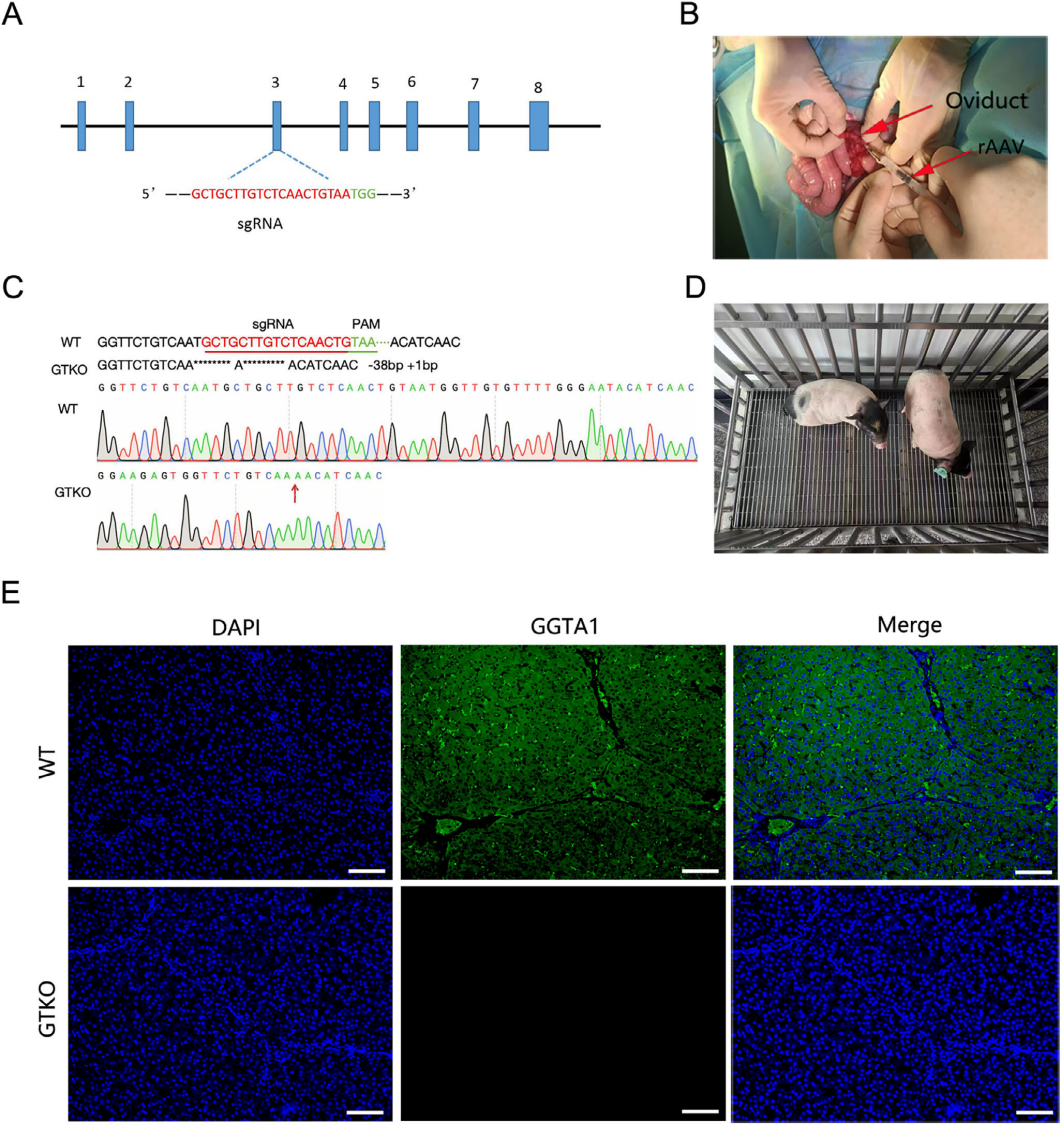
Generation of GGTA1 knockout pigs via oviduct injection. (A) Schematic representation of the GGTA1 gene structure and CRISPR/Cas9 sgRNA targeting site. (B) rAAV‐CRISPR/Cas9 oviduct injection surgery. (C) Sequencing results of gene‐edited pig fetuses produced by in vivo injection method. (D) Piglets born by the one‐step oviduct injection method. (E) Immunofluorescence of GGTA1 gene pig‐edited (GTKO) pig compared with WT pigs. Scale bar = 100 µm. GTKO, GGTA1 knockout; WT, wild‐type; sgRNA, single‐guide RNA; rAAV, recombinant adeno‐associated virus.

We demonstrated in a previous study that transgenic pigs can be obtained by injecting the rAAV‐CRISPR/Cas9 vector directly into the oviducts of pregnant sows to modify the embryos in vivo. In this study, a single‐guide RNA (sgRNA) was designed to target exon 3 of the *GGTA1* gene, with a 20 bp recognition sequence (5′‐GCTGCTTCTCAACTGTAA‐3′) followed by the PAM motif (TGG) (Figure 1A). scAAV6‐U1a‐spCas9 and scAAV6‐U6‐sgGGTA1‐CMV‐EGFP were mixed at a 1:1 ratio (3 × 10^10^ GC) and injected into the oviductal ampulla within 24 h after fertilization (Figure 1B). A total of seven pigs received the targeted rAAV delivery, and pregnancy was examined by ultrasound 30 days later. Fifteen piglets were born after 114 days of gestation, and ear tissue samples were collected for genotyping. As a result, one piglet was characterized by biallelic mutations, with a 38 bp deletion and a 1 bp insertion near the PAM sequence (Figure 1C). To establish a stable homozygous knockout colony, the GGTA1*^−/−^* founder animal was crossbred with wild‐type pigs, and homozygous offspring were selected. Repeated breeding cycles expanded the homozygous mutant population. Immunofluorescence analysis of liver tissue showed that GGTA1 was readily detectable in wild‐type pigs, but absent in knockout animals (Figure 1E).

### 2.2 Organoid Formation and Functional Characterization of Hepatocyte Organoids Co‐Cultured With R‐HUVEC

**FIGURE 2:**
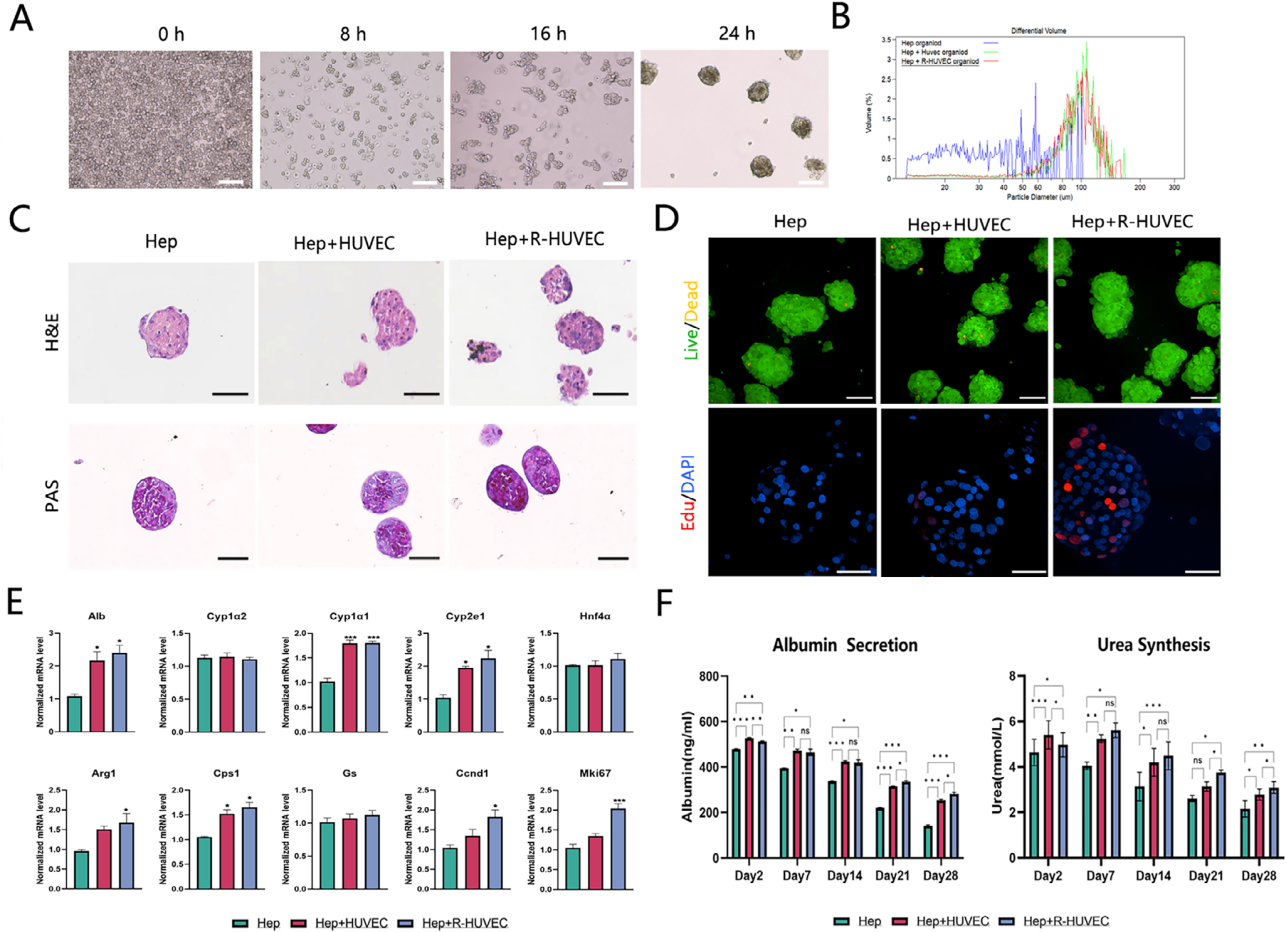
Generation of GGTA1 knockout pigs via oviduct injection. (A) Formation process within 24 h in four groups. Scale bar = 100 µm. (B) Diameter analysis of the organoids measured by Multisizer. (C) H&E and PAS staining of the organoids after 2 days of culture. Scale bar = 50 µm. (D) EdU staining of organoids. Scale bar = 50 µm. (E) Quantification of the gene expression of hepatocyte‐related genes and proliferation‐related genes (*p < 0.05, **p < 0.01, ***p < 0.001 compared with the Hep organoid, *n* = 3). (F) Albumin secretion and urea synthesis over 28 days in 3D cultures (*p < 0.05, **p < 0.01, ***p < 0.001, *n* = 3). H&E, hematoxylin and eosin staining; PAS, periodic acid–Schiff staining; EdU, 5‐ethynyl‐2′‐deoxyuridine; Hep organoid, hepatocyte organoid.

In order to generate functional liver organoids for BAL applications, we constructed hepatocyte organoids through co‐culture with endothelial cells. To first confirm the successful establishment of the RSPO1‐overexpressing HUVEC line (R‐HUVECs), we performed quantitative RT‐PCR and ELISA analysis. The results demonstrated a significant increase in *RSPO1* mRNA levels in R‐HUVECs compared to the wild‐type HUVEC control (Figure S1B). Furthermore, ELISA of the culture supernatant confirmed that R‐HUVECs successfully secreted high levels of RSPO1 protein, which was nearly undetectable in the control group (Figure S1C). These results verify the stable expression and secretory function of RSPO1, which is intended to provide a growth‐promoting niche for the hepatocytes. Subsequently, as shown in Figure 2A, the isolated hepatocytes gradually self‐assembled into compact organoids within 24 h. Particle size distribution (Figure 2B) confirmed the formation of uniform, cell‐dense aggregates. Histological analysis (Figure 2C) showed that the organoid structure in the Hep+R‐HUVEC group was enhanced, with improved glycogen storage capacity, as indicated by stronger hematoxylin‐eosin (H&E) and PAS staining. These results indicate better structural integrity and increased metabolic activity. Live/dead staining showed strong viability in all groups, with the Hep+R‐HUVEC organoids demonstrating higher cell survival. In addition, EdU incorporation assays revealed markedly higher proliferative activity in the R‐HUVEC co‐culture group (Figure 2D), showing enhanced cell renewal capacity. Molecular evaluation of liver‐specific markers, including Alb, Arg1, Cyp1a1, and CPS1, showed significantly higher expression in the Hep+R‐HUVEC group compared with the Hep group (Figure 2E), indicating enhanced hepatic identity. Furthermore, longitudinal analysis of liver function demonstrated significantly higher levels of albumin secretion and urea synthesis in the Hep+R‐HUVEC organoids during 28 days of culture (Figure 2F), confirming sustained metabolic activity and biosynthetic capacity. These results suggest that co‐culture with RSPO1‐overexpressing HUVECs not only accelerates organoid formation but also promotes hepatocyte maturation, viability, and long‐term functionality, providing a potent cellular unit for the BAL system.

### 2.3 Biochemical and Inflammatory Responses to Sequential NAL and BAL Therapy in ACLF Patients

**FIGURE 3:**
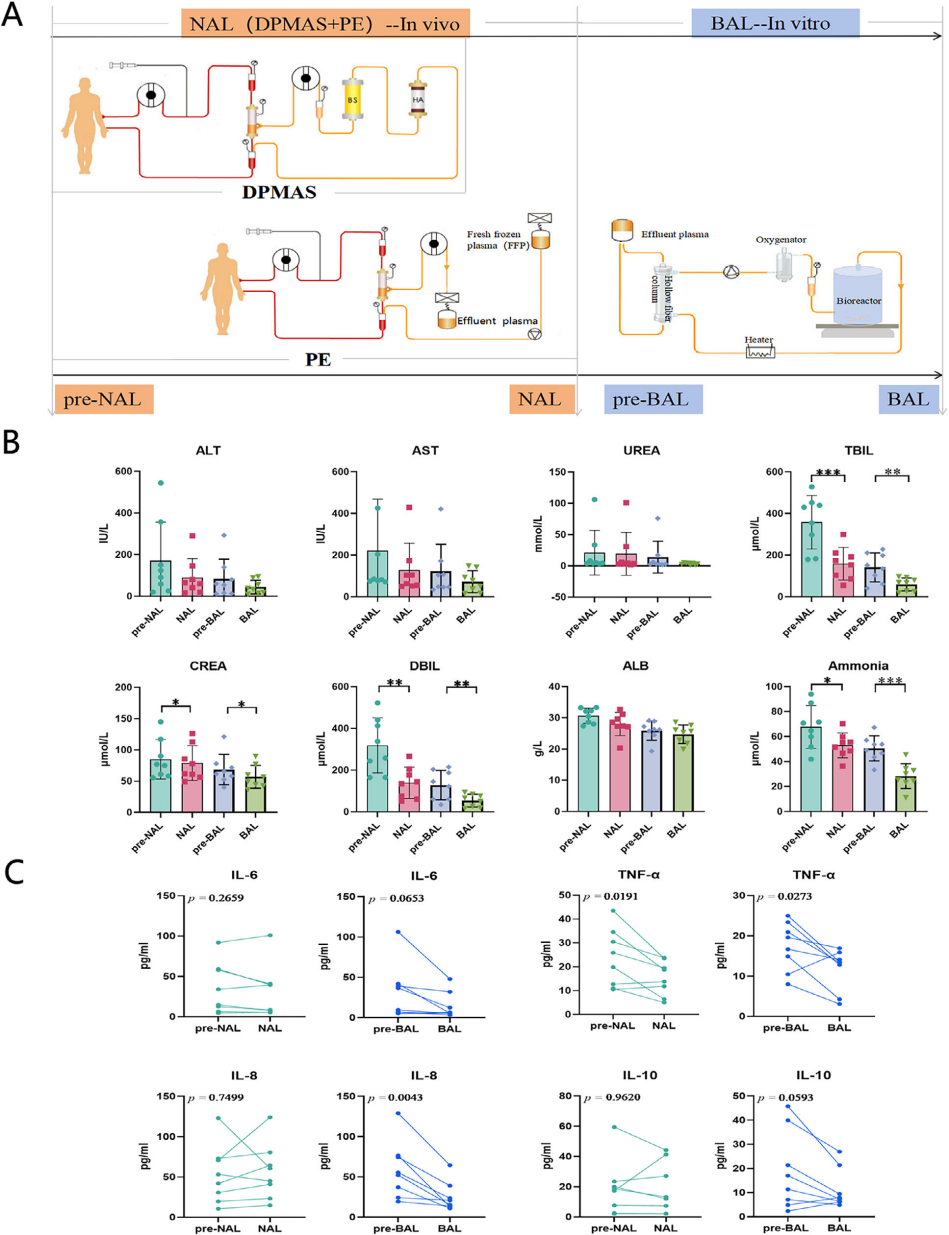
Effects of NAL and BAL treatment on liver function and inflammatory cytokines in ACLF patients. (A) Workflow of sequential artificial liver therapy. NAL consisted of in vivo DPMAS + PE, and the displaced plasma was subsequently treated by BAL in vitro (*p < 0.05, **p < 0.01, ***p < 0.001, *n* = 8). (B) Changes in biochemical indicators (ALT, AST, UREA, TBIL, CREA, DBIL, ALB, ammonia) before and after NAL and BAL treatment. (C) Inflammatory cytokine levels (IL‐6, IL‐8, IL‐10, and TNF‐α) before and after each treatment. Significant reductions were observed in TNF‐α (both NAL and BAL) and IL‐8 (BAL only). ACLF, acute‐on‐chronic liver failure; ALB, albumin; ALT, alanine aminotransferase; AST, aspartate aminotransferase; BAL, bioartificial liver; CREA, creatinine; DBIL, direct bilirubin; DPMAS, double plasma molecular adsorption system; IL, interleukin; NAL, non‐bioartificial liver; PE, plasma exchange; TBIL, total bilirubin; TNF‐α, tumor necrosis factor‐alpha.

To explore the differences in therapeutic efficacy between BAL and NAL support, we employed a sequential treatment approach. In clinical practice, patients underwent NAL therapy consisting of DPMAS combined with plasma exchange (PE). Plasma obtained during PE (pre‐BAL) was then subjected to BAL treatment in the laboratory (Figure 3A). This approach allowed comparison of biochemical parameters before and after each treatment using paired plasma samples from the same individuals. A total of eight ACLF patients were included in the study, comprising six males and two females, with a mean age of 46.8 years (range: 16 –70). Hepatitis B virus infection was the main etiology (6/8, 75%), with one patient having HBV combined with drug‐induced liver injury and another with HBV plus alcohol‐related liver disease. Baseline liver function was markedly impaired, as reflected by elevated TBIL levels (mean: 371.9 µmol/L, range: 179.5–528.0) and reduced ALB levels (mean: 29.3 g/L, range: 27.4–33.3). The median MELD score was 23 (range: 19–32) (Table 1).

**TABLE 1:** Baseline characteristics of the enrolled ACLF patients (*n* = 8).

ID	Age (years)	Sex	Etiology	ALT (U/L)	AST (U/L)	CREA (µmol/L)	TBIL (µmol/L)	ALB (g/L)	PT (s)	INR	MELD score
1	59	M	HBV	544.3	752.9	77.0	528.0	33.3	18.0	1.56	24
2	28	F	HBV+ drug‐induced	356.6	425.8	58.3	179.5	32.1	20.9	1.87	22
3	60	M	HBV	150.2	204.7	117.1	439.8	27.4	18.5	1.74	23
4	16	M	HBV	91.4	82.8	61.2	355.5	32.2	18.2	1.57	23
5	49	M	HBV	124.2	85.5	75.0	452.1	28.6	24.1	2.07	27
6	70	M	HBV+ post‐hepatectomy	25.9	75.3	145.1	297.6	28.0	24.9	2.36	32
7	60	M	HBV	58.5	76.9	55.5	182.7	32.3	20.8	1.79	22
8	42	M	HBV + alcohol	20.3	71.1	90.3	430.2	30.5	21.0	1.84	19

*Note*: Data are presented as individual patient values.

Abbreviations: ACLF, acute‐on‐chronic liver failure; ALB, albumin; ALT, alanine aminotransferase; AST, aspartate aminotransferase; CREA, creatinine; HBV, hepatitis B virus; INR, international normalized ratio; MELD, Model for End‐Stage Liver Disease; PT, prothrombin time; TBIL, total bilirubin.

Biochemical analysis revealed that ALT and AST levels showed a downward trend after both NAL and BAL treatment, but in some patients, statistical significance was not reached due to variability and enzyme–bilirubin dissociation (Figure 3B). After NAL treatment, TBIL values decreased significantly (from 353.2 ± 136.7 to 159.2 ± 78.22 µmol/L; DBIL: from 314.6 ± 137.4 to 139.5 ± 75.34 µmol/L). After BAL treatment, the values were further reduced (TBIL: 59.45 ± 32.32 µmol/L; DBIL: 54.14 ± 30.60 µmol/L, *p* < 0.001). Serum albumin (ALB) values remained almost stable during treatment (∼30 g/L), while serum creatinine (CREA) showed a downward trend after treatment, but due to baseline variability it did not reach statistical significance. Baseline ammonia levels in all ALF patients were elevated, consistent with hepatic encephalopathy. After NAL treatment, ammonia levels decreased significantly (*p* < 0.05), and further decreased after BAL treatment (*p* < 0.001). Most samples nearly reached normal levels (Figure 3B).

**FIGURE 4:**
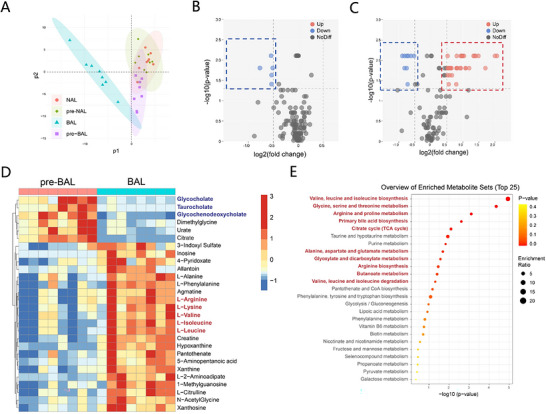
Targeted metabolomic analysis of plasma samples from NAL and BAL treatment groups. (A) PLS‐DA plot showing separation between pre‐ and post‐treatment groups. (B and C) Volcano plots showing differentially expressed metabolites after NAL and BAL. (D) Heatmap of significantly changed metabolites between pre‐BAL and BAL. (E) Pathway enrichment analysis (Top 25 pathways) after BAL. BAL, bioartificial liver; NAL, non‐bioartificial liver; PLS‐DA, partial least squares–discriminant analysis.

The inflammatory cytokines including IL‐6, IL‐8, interleukin‐10 (IL‐10), and TNF‐α were measured in plasma samples collected before and after treatment with BAL and NAL (Figure 3C). After NAL (*p* = 0.0191) and BAL (*p* = 0.0273) treatment, TNF‐α levels decreased significantly, indicating that both treatments could effectively reduce systemic inflammation, which is critical in ACLF management as it is a core driver of the syndrome's pathophysiology [1]. Similarly, IL‐8 levels decreased significantly after BAL treatment (*p* = 0.0043), confirming the anti‐inflammatory effect of the BAL system. IL‐6 levels decreased in both treatment groups, but the changes did not reach statistical significance (NAL: *p* = 0.2659; BAL: *p* = 0.0653). However, the downward trend suggests that it may contribute to reducing pro‐inflammatory signaling. As an anti‐inflammatory cytokine, IL‐10 did not show statistically significant changes with either treatment (NAL: *p* = 0.9620; BAL: *p* = 0.0593). However, a slight increase in the BAL group may indicate an enhanced anti‐inflammatory response.

### 2.4 Comparative Metabolomic Profiling Reveals Enhanced Detoxification and Amino Acid Restoration by BAL Versus NAL

To gain a more detailed understanding of the metabolic effects of AL support, targeted metabolomic analysis was conducted on blood samples collected during key treatment stages. Principal component analysis (PCA) revealed four distinct clusters: pre‐NAL, NAL, pre‐BAL, and BAL (Figure 4A). In particular, samples from BAL therapy showed the most pronounced separation along the first principal component (PC1), indicating that BAL therapy induced significant metabolic changes.

To quantify these changes, volcano plots were created to compare metabolites before and after treatment. In the NAL group, only a limited number of metabolites showed significant differences, whereas BAL treatment induced more extensive metabolite alterations (Figure 4B,C), with both up‐ and downregulated metabolites enriched in amino acid and bile acid metabolism pathways.

Hierarchical clustering analysis (HCA) confirmed that, compared with pre‐BAL samples, BAL treatment significantly altered the metabolic landscape (Figure 4D). The upregulated metabolites included branched‐chain amino acids (valine, leucine, isoleucine), arginine, lysine, sarcosine, urate, etc., which are intermediates of nitrogen and energy metabolism. In contrast, bile acids (e.g., glycocholate and taurocholate) and uremic toxins (e.g., indoxyl sulfate) were significantly decreased after BAL treatment, consistent with enhanced detoxification. A complete list of significantly altered metabolites, with fold changes and *p*‐values, can be found in Table S2.

Pathway enrichment analysis identified multiple significantly affected pathways post‐BAL, including valine, leucine, and isoleucine biosynthesis, glycine and serine metabolism, arginine and proline metabolism, primary bile acid biosynthesis, and the tricarboxylic acid (TCA) cycle (Figure 4E).

These findings suggest that BAL therapy not only contributes to the removal of toxic metabolites but may also facilitate partial restoration of amino acid and energy metabolism. Collectively, these results highlight the metabolic remodeling associated with BAL treatment and underscore its superior capacity for systemic detoxification compared to NAL.

### 2.5 Targeted Lipidomics Reveals Distinct Metabolic Signatures of BAL Versus NAL Treatment

To investigate how biological and non‐biological AL treatments (BAL and NAL) differently influence lipid metabolism, we performed targeted lipidomics on plasma samples collected before and after treatment. Using LC‐MS, 1243 lipid species were quantified, and analysis focused on those showing notable fold changes and high VIP scores.

**FIGURE 5:**
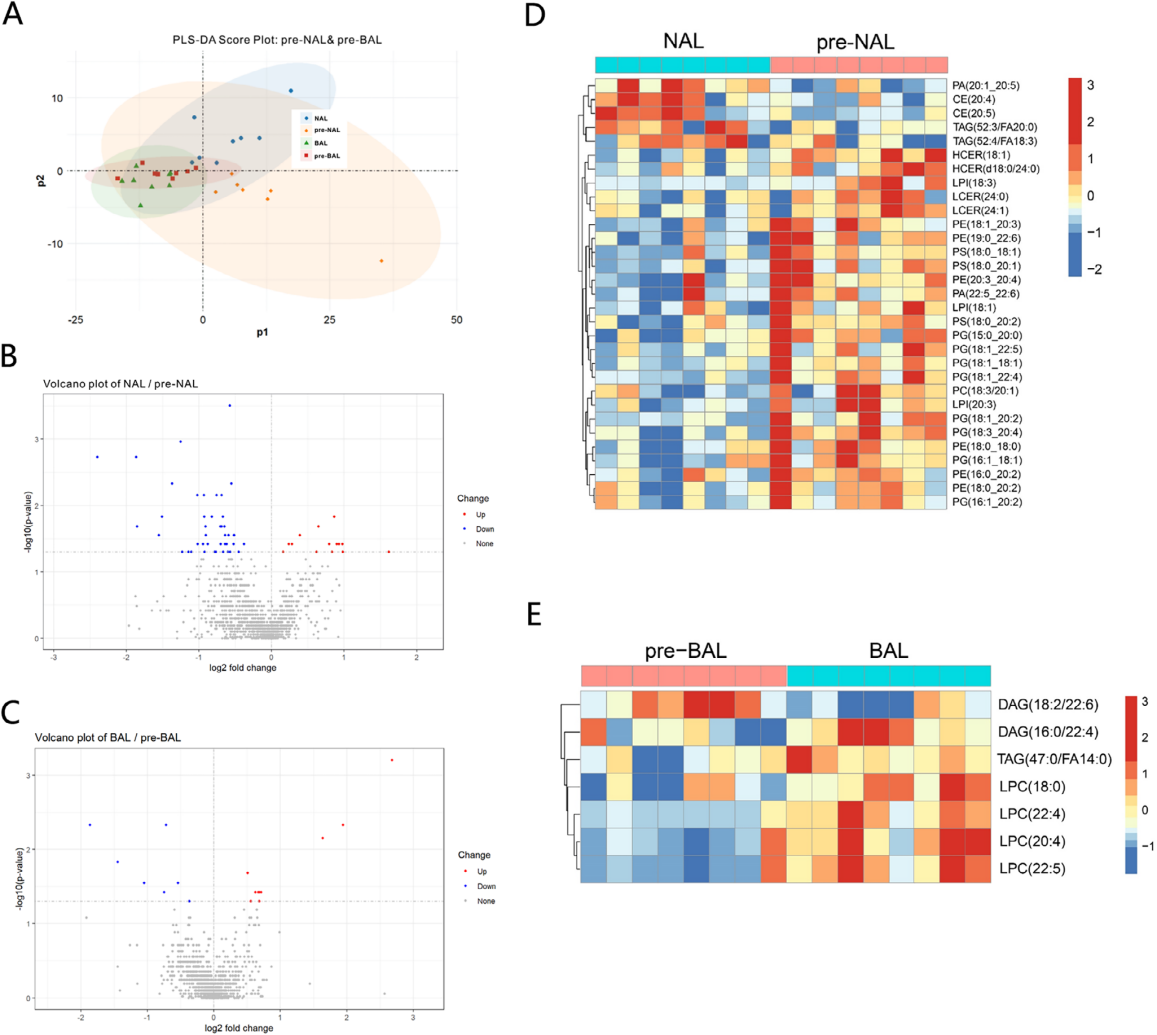
Comparison of lipidomic changes induced by NAL and BAL treatment. (A) PLS‐DA score plot showing separation among pre‐NAL, NAL, pre‐BAL, and BAL samples. (B and C). Volcano plot comparing lipid changes after NAL and BAL (D and E). Heatmap of top differential lipids between pre‐NAL and NAL groups and pre‐BAL and BAL groups. BAL, bioartificial liver; NAL, non‐bioartificial liver; PLS‐DA, partial least squares–discriminant analysis.

PLS‐DA showed a moderate separation between pre‐ and post‐treatment samples (Figure 5A), indicating that both interventions triggered changes in the plasma lipidome. Yet, the patterns differed strikingly. Volcano plots revealed that NAL treatment altered a larger number of lipid species overall (Figure 5B,C). This suggests a broad, but somewhat unspecific, disturbance of lipid metabolism.

Hierarchical clustering provided further insight. NAL induced widespread shifts across multiple lipid classes, including increases in phosphatidic acids (PAs), cholesteryl esters (CEs), and highly unsaturated phospholipids such as phosphatidylethanolamines (PEs), phosphatidylserines (PSs), and phosphatidylglycerols (PGs) (Figure 5D). These changes might reflect partial activation of lipid pathways and early resolution of inflammation. However, the responses varied considerably between individuals, giving a diffuse and heterogeneous pattern.

In contrast, BAL elicited a more precise and consistent response. Post‐treatment, lysophosphatidylcholine (LPC) species (18:0, 20:4, 22:4, 22:5) and specific diacylglycerols (DAG 18:2/22:6, 16:0/22:4) increased markedly (Figure 5E). These lipids are closely linked to membrane remodeling, anti‐inflammatory signaling, and cellular energy regulation. Clustering analysis showed that BAL‐treated samples grouped tightly, with less variability between patients, suggesting a coordinated metabolic adjustment.

Taken together, NAL caused broader lipidomic perturbations, but BAL promoted more selective, biologically meaningful changes. The results imply that BAL may better support lipid homeostasis, fine‐tuning pathways involved in membrane integrity and liver recovery.

## 3 Discussion

The BAL system has emerged as a pivotal therapeutic modality, capable of furnishing transient hepatic support for patients with liver failure, thereby mitigating the pathological burden and serving as a vital bridge to native liver regeneration or transplantation. Nevertheless, its clinical translation has been consistently hindered by fundamental bottlenecks pertaining to the safety of cell sources and the preservation of robust cellular functionality in vitro. Although advancements have been documented involving transdifferentiated cells and GMP‐grade induced hepatocytes, these methodologies are frequently constrained by intricate and protracted large‐scale manufacturing protocols, and their long‐term functional stability necessitates further rigorous validation [8, 9, 14].

In the present study, a systematic strategy centered on primary porcine hepatocytes is proposed, thereby circumventing the inherent complexities and financial burdens associated with cellular reprogramming. This investigation directly addresses the primary limitations of utilizing primary cells—specifically regarding safety and functionality—via a dual‐pronged approach.

First, to address the critical issue of immunogenicity, CRISPR/Cas9 gene‐editing technology was employed to generate GGTA1 knockout Bama miniature pigs. This strategy abrogates the expression of the α‐1,3‐galactose antigen, a key xenoantigen, thereby mitigating the risk of hyperacute immune rejection [30]. As confirmed in previous work by our group, this provides a safer and more scalable source of primary cells for xenotransplantation applications [31].

Second, to overcome the challenge of limited in vitro longevity and functional decline, an advanced R‐HUVEC co‐culture system was developed based on previous findings [32]. By engineering HUVECs to secrete the RSPO1 protein, the Wnt/β‐catenin signaling pathway was activated, achieving dual regulation of liver organoid proliferation and function [21, 33]. This was corroborated by our results, wherein EdU staining revealed a significant increase in hepatocyte proliferation, and the organoids maintained high levels of albumin secretion and urea synthesis throughout a 28‐day culture period.

Beyond the secretion of RSPO1, the multifaceted supportive role of HUVECs in maintaining hepatic functionality warrants further consideration. Recent studies have underscored that endothelial cells serve as an essential component of the hepatic niche, providing not only structural scaffolding but also a complex array of angiocrine factors that govern hepatocyte maturation and metabolic homeostasis [34, 35]. In our organoid model, the co‐culture of hepatocytes with R‐HUVECs likely facilitates critical cell–cell interactions and paracrine signaling crosstalk, which synergistically enhances the biosynthetic and detoxification capacities of primary porcine hepatocytes. Thus, the integration of engineered HUVECs in our BAL system represents a holistic strategy to simulate the native liver microenvironment, ensuring sustained performance during clinical application.

The use of hepatocyte‐endothelial co‐cultured organoids in this study aligns with current tissue engineering trends advocating for multicellular aggregates to restore hepatic function [36]. Such 3D configurations not only ensure superior cell survival but also enhance antioxidant capacity and liver regeneration through inflammatory modulation [37]. Recent studies have further optimized these systems by incorporating 3D‐printed scaffolds with antioxidant nanozymes or utilizing spheroid‐derived extracellular vesicles to provide pro‐angiogenic support [38, 39]. Most importantly, the results of peers mirror the findings that vascularized 3D‐printed liver models can activate Wnt signaling to drive hepatic maturation and effectively rescue liver failure by suppressing local inflammation, providing strong theoretical and empirical support for our BAL strategy [40].

This integrated approach, which combines genetic engineering with an advanced co‐culture strategy, provides a safer, more stable, and potentially more scalable and cost‐effective cell source for clinical BAL applications.

While the clinical efficacy of BAL has been demonstrated in numerous studies, its underlying molecular mechanisms have remained largely unelucidated. Through a systematic metabolomic analysis of plasma from ACLF patients, this study systematically characterized the unique therapeutic mechanism of the BAL system, differentiating it from conventional NAL therapy.

Our metabolomic results indicated that the primary function of NAL treatment is physical adsorption, which exerts a limited and non‐specific effect on the plasma metabolome, with minimal improvement observed in amino acid and lipid metabolism. In contrast, the core function of BAL treatment was identified as active biological regulation. Following BAL treatment, metabolic pathways pertinent to protein synthesis and energy metabolism, such as the branched‐chain amino acid (BCAA) pathway, were found to be significantly upregulated, evidenced by the elevated expression of metabolites including L‐leucine and L‐isoleucine. This suggests an improvement in overall protein synthesis and energy metabolism. Concurrently, a decrease in citrate levels within the TCA cycle was observed, indicative of a partial recovery of hepatic energy metabolism [15]. Furthermore, the significant reduction in bile acid and uric acid levels reflected the enhanced detoxification capacity of the system.

In our study, although it is not statistically significant, there is a rising trend in the levels of IL‐10 following BAL treatment (*p* = 0.0593). IL‐10 is a classical anti‐inflammatory cytokine that plays a pivotal role in maintaining immunological homeostasis. In the context of ACLF, the body often undergoes an intense systemic inflammatory response syndrome (SIRS), which can lead to catastrophic multi‐organ failure if left unchecked. The upward trend of IL‐10 in the BAL group suggests that the BAL system may actively facilitate a shift from a hyper‐inflammatory state toward an anti‐inflammatory, pro‐resolution phase. This is consistent with recent findings suggesting that therapeutic interventions in liver failure, such as cell‐based therapies, can attenuate acute injury by promoting a switch toward an anti‐inflammatory phenotype [41, 42]. By boosting the anti‐inflammatory milieu, our BAL system potentially protects the remaining hepatocytes from secondary damage caused by the “cytokine storm,” thereby creating a more favorable microenvironment for liver regeneration and functional recovery.

Targeted lipidomics analysis provided additional mechanistic insights. After BAL treatment, the levels of key lipid molecules, including LPCs and DAGs, were significantly upregulated. This observation suggests that BAL treatment confers beneficial effects on the restoration of lipid metabolic balance, the promotion of cell membrane repair, and the provision of cellular energy, which may also contribute to the regulation of inflammation. Collectively, these findings indicate that the BAL system functions not merely as a passive detoxification tool but as an active biological regulator that modulates systemic metabolism to support the recovery of liver function [28, 29, 43].

A critical consideration in our study design is whether the “pre‐BAL” baseline—representing the plasma state after initial NAL treatment—affects the evaluation of BAL efficacy. NAL procedures, such as DPMAS and PE, provide a robust “physical clearance” of accumulated toxins, including bilirubin and ammonia, effectively resetting the systemic biochemical environment. While this more refined baseline might theoretically narrow the margin for further detoxification, our multi‐omics results demonstrate that BAL treatment achieves an “active metabolic enhancement” that transcends simple physical removal. Unlike the passive and non‐selective processes of NAL, the BAL system functions as a biological regulator. It not only further reduces toxic burdens but also actively restores metabolic homeostasis by synthesizing essential proteins and modulating complex lipid profiles, such as the upregulation of specific LPC species. This distinction underscores that BAL is not merely an adjunct to physical therapy but a vital biological intervention that provides indispensable metabolic support for the failing liver.

A standout finding in our lipidomic profiling was the significant upregulation of various LPC species following BAL treatment (Figure 5). This metabolic shift highlights a fundamental functional divergence between the biological support of BAL and the physical clearance of NAL. In clinical ACLF, the characteristic depletion of plasma LPC levels correlates strongly with disease severity and poor prognosis, reflecting both intense systemic inflammation and impaired hepatic biosynthetic capacity [44]. Recent studies have confirmed that LPC levels are significantly positively correlated with the prognosis of patients with acute liver failure (ALF). Specifically, LPCs have been shown to suppress hyperinflammation and induce a pro‐resolution macrophage phenotype by regulating monocyte/macrophage immune checkpoints [45]. By actively restoring these essential lipid species, our BAL system provides an “active biological repair” mechanism. This transcends the passive physical filtration of NAL, offering a more comprehensive metabolic support that is essential for the recovery of the failing liver.

While this study establishes a robust technical and theoretical foundation for the clinical translation of BAL technology, it is subject to certain limitations. Primarily, the experiments involving plasma from ACLF patients were conducted ex vivo, and the clinical translatability of these findings must be validated through subsequent in vivo animal studies and human clinical trials. Second, the relatively small sample size and the etiological heterogeneity of the patient cohort may influence the interpretation of the statistical results. Future investigations will be required to expand the sample size and perform stratified analyses to mitigate the impact of inter‐individual variability.

Furthermore, although bioreactor optimization was not a primary focus of this study, it remains a critical aspect of BAL development. Our previous work utilizing the Spheroid Reservoir Bioartificial Liver (SRBAL) in a rhesus macaque model of ALF has already demonstrated promising outcomes [7]. The design of the bioreactor presents a significant challenge, necessitating further optimization to balance the delivery of oxygen and nutrients while minimizing mechanical shear forces on the cells. These findings provide important directions for our future research endeavors. Furthermore, while the *GGTA1* knockout presented herein is a critical step for mitigating hyperacute rejection, the future of xenotransplantation cell sources will likely involve more complex, multi‐gene editing strategies to achieve a higher degree of “humanization” and long‐term functional compatibility [30, 46].

Accordingly, future research will be directed toward several key areas. First, the optimized cellular strategy developed in this study will be applied to animal models for comprehensive in vivo validation. Second, the integration of multi‐omics techniques, including proteomics and transcriptomics, will be employed to more comprehensively elucidate the mechanisms of action of the BAL system. Furthermore, the successful clinical translation of such organoid‐based systems necessitates rigorous pathological evaluation to ensure cellular fidelity and functional consistency, a critical requirement for precision medicine applications [47]. Finally, large‐scale, multi‐center clinical trials will be essential to fully evaluate the safety and efficacy of this technology in a clinical setting.

In conclusion, this study demonstrates that the BAL system not only provides rapid detoxification but also actively regulates systemic metabolism at a molecular level to support the recovery of hepatic function. Although these results represent a significant advancement, large‐scale clinical trials and more in‐depth mechanistic studies are imperative to fully assess the safety and efficacy of this system for future clinical application.

## 4 Materials and Methods

### 4.1 Generation and Identification of Genetically Engineered Porcine Liver Cells

To obtain a cell source with reduced immunogenicity, recombinant adeno‐associated virus (rAAV)‐mediated CRISPR/Cas9 technology was employed for the targeted knockout of the GGTA1 gene in primary porcine hepatocytes. Initially, single guide RNA (sgRNA) target sequences specific to exon 3 of the *GGTA1* gene were designed using an online sequence analysis tool (http://crispor.tefor.net/). These sgRNA sequences were subsequently ligated into the PX458 CRISPR/Cas9 expression vector and transfected into porcine embryonic fibroblasts to assess their cleavage efficiency. The sgRNA sequence demonstrating the highest efficacy was then packaged into a self‐complementary AAV (scAAV) vector, while the spCas9 was packaged into a single‐stranded AAV (ssAAV) vector.

Following the generation of viral vectors, in vivo gene editing was performed using a one‐step oviduct injection method. The estrous cycles of Bama miniature pigs were monitored, and within 24 h of natural mating, a 1:1 mixture of scAAV6‐U6‐sgGGTA1‐CMV‐EGFP and ssAAV6‐Ula‐spCas9 was injected into the ampullary region of the oviduct.

Upon the birth of the piglets, ear tissue samples were collected, and genomic DNA was extracted using a commercial DNA extraction kit. Genotyping of the target locus was performed via Sanger sequencing. For samples that exhibited heterozygous peaks in the sequencing chromatogram, TA cloning followed by sequencing was utilized to identify the specific mutation types. To further confirm the successful gene knockout, immunofluorescence analysis was conducted on liver tissues from the genetically engineered pigs to verify the abrogation of GGTA1 protein expression.

### 4.2 Construction and Functional Evaluation of R‐HUVEC Co‐Cultured Liver Organoids

To overcome the challenges of limited proliferation and functional maintenance of primary porcine hepatocytes in vitro, a three‐dimensional co‐culture strategy was developed, incorporating HUVECs engineered to overexpress the RSPO1 protein.

First, primary hepatocytes (Hep) were isolated from Bama miniature pigs using a three‐step in situ collagenase perfusion method. Subsequently, the R‐HUVEC cell line was established. The lentiviral vector encoding RSPO1 (HBLV‐h‐RSPO1‐3xflag‐ZsGreen‐PURO), constructed by Hanheng Company, was used to transduce HUVEC cells. Viral transduction efficiency was evaluated via fluorescence microscopy, and puromycin selection was performed to obtain a stably transduced R‐HUVEC cell line. The successful overexpression of RSPO1 was then confirmed at the mRNA and protein levels using qRT‐PCR and ELISA, respectively.

For co‐culture experiments, isolated primary hepatocytes were mixed with R‐HUVEC cells at a 50:1 ratio and were cultured in a three‐dimensional shaking system to facilitate the formation of liver organoids. To evaluate the functional benefits of this co‐culture system, three experimental groups were established: Hep, Hep+HUVEC, and Hep+R‐HUVEC.

The functional performance of the resulting liver organoids was evaluated through multiple assays. Cell proliferation was examined using EdU staining, and the expression of proliferation‐associated genes such as CCND1 and MKi67 was quantified by qRT‐PCR. Hepatic function was assessed by measuring albumin secretion in the culture supernatant using an ELISA kit and by determining urea synthesis with a QuantiChrom Urea Assay Kit. Additionally, the expression of hepatocyte‐specific genes, including ALB, HNF4A, and CYP1A1, was analyzed by qRT‐PCR to further confirm hepatocyte functionality.

### 4.3 Preclinical Study of the Bioartificial Liver System in ACLF Patient Plasma

To evaluate the preclinical efficacy and therapeutic mechanisms of the BAL system, an ex vivo plasma purification study was conducted using plasma obtained from ACLF patients. The study protocol was approved by the Institutional Review Board of West China Hospital, Sichuan University (Approval No.: 2019–204).

#### 4.3.1 Clinical Patient Enrollment and Plasma Sample Collection

Eight ACLF patients undergoing NAL treatment, comprising DPMAS combined with half‐volume PE, were enrolled in this study.

- Study population

Patients with ACLF who were hospitalized at the Center of Infectious Diseases, West China Hospital, Sichuan University, between March 1 and June 30, 2023, and were scheduled to receive AL support therapy were prospectively enrolled in this study.

- Inclusion criteria

Participants meeting all of the following criteria were included:

- age ≥ 16 years;
- diagnosis of ACLF according to the Asia‐Pacific Association for the Study of the Liver (APASL) consensus recommendations (2022 edition);
- planned to receive first‐time NAL support therapy, specifically dDPMAS combined with PE.

- Exclusion criteria

Patients meeting any of the following conditions were excluded:

- failure to meet the APASL diagnostic criteria for ACLF;
- previous receipt of any form of AL support therapy;
- presence of other disease conditions that, in the investigator's judgment, could substantially alter plasma metabolic profiles, such as active hematological malignancies or uncontrolled systemic autoimmune diseases;
- inability to complete blood sample collection according to the study protocol, or plasma samples deemed unsuitable for analysis due to severe hemolysis, lipemia, or other quality‐related issues.

The DPMAS procedure utilized a Jafron DX‐10 blood purification machine connected to a plasma separator, a bilirubin adsorption column (BS330), and a plasma perfusion column (HA330‐II) for physical detoxification. Plasma collected during the exchange procedure was reserved for the subsequent ex vivo BAL treatment. The BAL system consisted of a bioreactor embedded with liver organoids to simulate hepatic metabolic functions. Within this system, patient plasma was perfused through a hollow fiber column with a 65 kDa molecular weight cutoff, allowing for the diffusion of small‐molecule water‐soluble toxins as well as medium‐ and large‐molecule protein‐bound toxins into the dialysate. The dialysate was circulated at 300 mL/min, delivering the toxins to the bioreactor for metabolism by the hepatocyte organoids. Throughout the procedure, the dialysate was monitored and regulated by temperature, oxygen, and pH probes to ensure the viability and biological activity of the organoids. Four sets of plasma samples were collected for analysis: pre‐NAL treatment (pre‐NAL group), post‐NAL treatment (NAL group), pre‐BAL treatment (pre‐BAL group), and post‐BAL treatment (BAL group). Subsequently, biochemical analyses, including measurements of blood ammonia, bilirubin, transaminases, and inflammatory factors, were performed. Blood ammonia levels were quantified within 30 min of collection using a Nanjing Jiancheng blood ammonia test kit.

#### 4.3.2 Targeted Metabolomics and Targeted Lipidomics Analysis

To evaluate the effects of different treatment modalities on plasma metabolites, targeted metabolomics and targeted lipidomics analyses were performed on the four sets of plasma samples. Metabolite quantification was carried out using liquid chromatography‐mass spectrometry. The resulting data were processed using multivariate statistical methods, including PCA, partial least squares‐discriminant analysis (PLS‐DA), and orthogonal partial least squares‐discriminant analysis (OPLS‐DA), to quantify differential metabolites. These analyses were also used to evaluate changes in metabolites and metabolic pathways associated with the recovery of liver function. Pathway analysis, based on the Kyoto Encyclopedia of Genes and Genomes (KEGG) Human database, was conducted to elucidate the biological significance of the observed metabolic changes.

#### 4.3.3 Statistical Analysis

All experimental data, excluding omics data, were presented as the mean ± standard deviation (mean ± SD). Statistical comparisons were performed using Student's *t*‐tests and one‐way analysis of variance (ANOVA). A *p*‐value of less than 0.05 was considered to be statistically significant. All statistical analyses were conducted using GraphPad Prism 8.5 software.

## Author Contributions

Yuting He and Yang Deng wrote the paper and performed the experiments and the statistical analysis. Qin Liu, Xinglong Zhu, Yanyan Zhou, Mengyu Gao, and Wanliu Peng performed the genetic editing of pigs and isolation of primary pig hepatocytes. Qin Liu, Yuting He, and Yanyan Zhou conducted histopathological experiments such as H&E, PAS, and fluorescence staining. Yang Deng, Qin Liu, Yuting He, and Lang Bai performed plasma exchange experiments and carried out biochemical assays on patient samples. Yang Deng and Yuting He performed lipidomics analysis. Lang Bai, Ji Bao, and Xinglong Zhu reviewed and edited the manuscript. Lang Bai and Ji Bao designed the research, which provided financial support for the study. All authors have read and approved the final manuscript.

## Funding

This work was supported by the National Natural Science Foundation of China (grant numbers 82270662 and 82070640), the Technology Innovation Project of Chengdu New Industrial Technology Research Institute (grant numbers 2018‐CY02‐00046‐GX), and the 1.3.5 Project for Disciplines of Excellence, West China Hospital, Sichuan University (grant number ZYJ:C21014).

## Ethics Statement

The animal study protocol was approved by the Institutional Animal Care and Use Committee (IACUC) of the Animal Experiment Center of Sichuan University (No. 202089A, March 23, 2020). The clinical study involving plasma from patients with acute‐on‐chronic liver failure (ACLF) was approved by the Ethics Committee of West China Hospital, Sichuan University (Approval No.: 2019–204). Written informed consent was obtained from all participants.

## Conflicts of Interest

The authors declare no conflicts of interest.

## Supporting information




**Supporting Table 1**: Primer sequences of the genes.
**Supporting Table 2**: Significantly altered metabolites before and after bioartificial liver (BAL) treatment.
**Supporting Figure 1**: Establishment of R‐HUVEC cells overexpressing RSPO1 protein.
*RSPO1* gene was successfully transfected into HUVEC. Brightfield imaging (left), fluorescence (middle) and merge (right) image of R‐HUVEC. Scale bar = 50 µm.
Quantifcation of the gene expression by RT‐qPCR of RSPO1. Data were normalized to the expression of HUVEC (**p<0.01, n = 3).
The expression of RSPO1 protein was detected by ELISA (***p<0.001, n = 3).

**Supporting File 1**: mco270795‐sup‐0002‐Data.xlsx

## Data Availability

The raw metabolomics data generated in this study are provided as Supporting Information associated with this article. Additional repository information will be updated prior to final publication.
