# Supplementary material for: Bioartificial Livers Developed From Gene‐Edited Pig Hepatocyte Organoids Improve Amino Acid and Lipid Profiles in the Plasma of Patients With Liver Failure
Source: MedComm (2020). 2026 May 31;7(6):e70795. doi: 10.1002/mco2.70795 (PMC13239287; doi:10.1002/mco2.70795)
Supplement: Supplementary file 1 — Supporting Table 1: Primer sequences of the genes. Supporting Table 2: Significantly altered metabolites before and after bioartificial liver (BAL) treatment. Supporting Figure 1: Establishment of R‐HUVEC cells overexpressing RSPO1 protein. RSPO1 gene was successfully transfected into HUVEC. Brightfield imaging (left), fluorescence (middle) and merge (right) image of R‐HUVEC. Scale bar = 50 µm. Quantifcation of the gene expression by RT‐qPCR of RSPO1. Data were normalized to the expression of HUVEC (**p<0.01, n = 3). The expression of RSPO1 protein was detected by ELISA (***p<0.001, n = 3). [file MCO2-7-e70795-s001.docx]

**Title:** **Bioartificial livers developed from gene-edited pig hepatocyte organoids improve amino acid and lipid profiles in the plasma of patients with liver failure**

**Running Title: Organoid-Derived BAL Modulates Plasma Metabolism**

Yuting He^1, 2, #^, Yang Deng^1, #^, Xinglong Zhu^1^, Mengyu Gao^3^, Qin Liu^1^, Wanliu Peng^1^, Yanyan Zhou^1^, Lang Bai^4,^ *, Ji Bao^1,^ *

^1^ Department of Pathology, Institute of Clinical Pathology, Key Laboratory of Transplant Engineering and Immunology, NHC, West China Hospital, Sichuan University, Chengdu 610041, Sichuan Province, China

^2^ Department of Pathology, West China Second University Hospital, Sichuan University, Chengdu, 610041, Sichuan Province, China; Key Laboratory of Birth Defects and Related Diseases of Women and Children (Sichuan University), Ministry of Education, Chengdu, Sichuan, China.

^3^ Department of Medical genetics/Prenatal Diagnostic Center, West China Second University Hospital, Sichuan University, Chengdu, Sichuan, China; Key Laboratory of Birth Defects and Related Diseases of Women and Children (Sichuan University), Ministry of Education, Chengdu, Sichuan, China.

^4^ Center of Infectious Diseases, West China Hospital, Sichuan University, Chengdu 610041, Sichuan Province, China

^#^Yuting He and Yang Deng contributed equally to the work and are co-first authors.

***Corresponding author:**

Lang Bai, PhD., Chief Physician, Center of Infectious Diseases, West China Hospital of Sichuan University, No. 37, Guoxue Alley, Wuhou District, Chengdu, Sichuan 610041, Sichuan Province, China. pangbailang@163.com. Telephone: +86-18980602254 Fax: +86-28-85164033.

Ji Bao, Ph.D., Professor, Department of Pathology, Institute of Clinical Pathology, Key Laboratory of Transplant Engineering and Immunology, NHC, West China Hospital, Sichuan University, No. 37, Guoxue Alley, Wuhou District, Chengdu 610041, Sichuan Province, China. [baoji@scu.edu.cn](mailto:baoji@scu.edu.cn). Telephone: +86-18980606618 Fax: +86-28-85164033.

**Supplement materials**

Table S1 Primer sequences of the genes.

| Gene | Abbr. | Forward Primers (5'-3') | Reverse Primers (5'-3') |
| --- | --- | --- | --- |
| Albumin | ALB | CCCTGTTGCTTTATGCGCTG | TATCAGCAGCTTGGCAGCAT |
| Arginase 1 | ARG1 | ACCTGACTGTGTCTTCCGTTC | GAGCTCCGATGATCCCGATG |
| Cyclin D1 | CCND1 | TCCAGGGTGATCAGGTGTGA | GGCGCCTCAAATGTTCACG |
| Carbamoyl-phosphate synthase 1 | CPS1 | AGGTCATCAAGGCAGAACGG | GGTAGCCAATGGTGTCTGCT |
| Cytochrome P450 family 1 subfamily A member 1 | CYP1A1 | ATCCAGAAGTCCCGTAGGTG | CCCTTCGTCCTCCCACAGTA |
| Cytochrome P450 family 1 subfamily A member 2 | CYP1A2 | GCCAATCTGATAGGGGTGATGT | ACTCTTCAGGCCTTTGGGGA |
| Cytochrome P450 family 2 subfamily E member 1 | CYP2E1 | GCACATCCACAGTAGCTGGAA | CAGGTGTCCTTGTGCCAATTC |
| Hepatocyte nuclear factor 4 alpha | HNF4A | AATCCTCATCCTGGCACTGC | GCGGTCGTTGATGTAATCCT |
| Glyceraldehyde 3-phosphate dehydrogenase | GAPDH | CCCCCTCAAGGGCATCCTG | GGTCCACCACCCTGTTGCTGTA |
| Glutamate-ammonia ligase | GLUL | CCTTCGTATTCCTGCTCATCCC | AGCTTGGACTTTCTCGCCCT |
| Marker of proliferation Ki-67 | MKI67 | GCTCCAGACAAGGGAATGTC | TGGGATTTTCGGCTCCATCC |
| R-spondin1 | RSPO1 | GTGCTACATGCCCCTGTG | GCCTCCCTTCCTCCTCTT |

Table S2 Significantly Altered Metabolites Before and After bioartificial liver (BAL) Treatment.

| **Compound** | **KEGG** | **pvalues** | **foldchange** | **OPLSDA_VIP** |
| --- | --- | --- | --- | --- |
| Urate | C00366 | 0.008 | 0.540 | 1.099 |
| Glycocholate | C01921 | 0.016 | 0.567 | 1.073 |
| Taurocholate | C05122 | 0.008 | 0.595 | 1.034 |
| Dimethylglycine | C01026 | 0.016 | 0.595 | 1.019 |
| Glycochenodeoxycholate | C05466 | 0.008 | 0.614 | 1.308 |
| Citrate | C00327 | 0.008 | 0.651 | 1.302 |
| L-Alanine | C00041 | 0.008 | 1.440 | 1.071 |
| L-Phenylalanine | C00079 | 0.016 | 1.486 | 1.133 |
| L-Arginine | C00062 | 0.023 | 1.499 | 1.115 |
| L-Valine | C00183 | 0.016 | 1.516 | 1.069 |
| Agmatine | C00179 | 0.016 | 1.535 | 1.141 |
| 3-Indoxyl Sulfate | C05362 | 0.016 | 1.569 | 1.017 |
| L-Lysine | C00047 | 0.023 | 1.582 | 1.058 |
| 4-Pyridoxate | C00847 | 0.008 | 1.754 | 1.080 |
| Xanthosine | C01762 | 0.016 | 1.756 | 1.050 |
| 1-Methylguanosine | C16137 | 0.016 | 1.765 | 1.219 |
| L-Citrulline | C00327 | 0.016 | 1.886 | 1.163 |
| Pantothenate | C00864 | 0.016 | 1.915 | 1.011 |
| L-Isoleucine | C00407 | 0.008 | 1.944 | 1.220 |
| L-Leucine | C00123 | 0.008 | 1.962 | 1.261 |
| Xanthine | C00385 | 0.008 | 2.050 | 1.143 |
| N-AcetylGlycine | C02642 | 0.016 | 2.126 | 1.002 |
| 5-Aminopentanoic acid | C00431 | 0.008 | 2.516 | 1.046 |
| Creatine | C00300 | 0.008 | 2.645 | 1.316 |
| Hypoxanthine | C00262 | 0.008 | 2.804 | 1.269 |
| Allantoin | C01551 | 0.016 | 3.255 | 1.019 |
| L-2-Aminoadipate | C00311 | 0.008 | 3.999 | 1.211 |
| Inosine | C00294 | 0.008 | 4.186 | 1.069 |

Figure S1


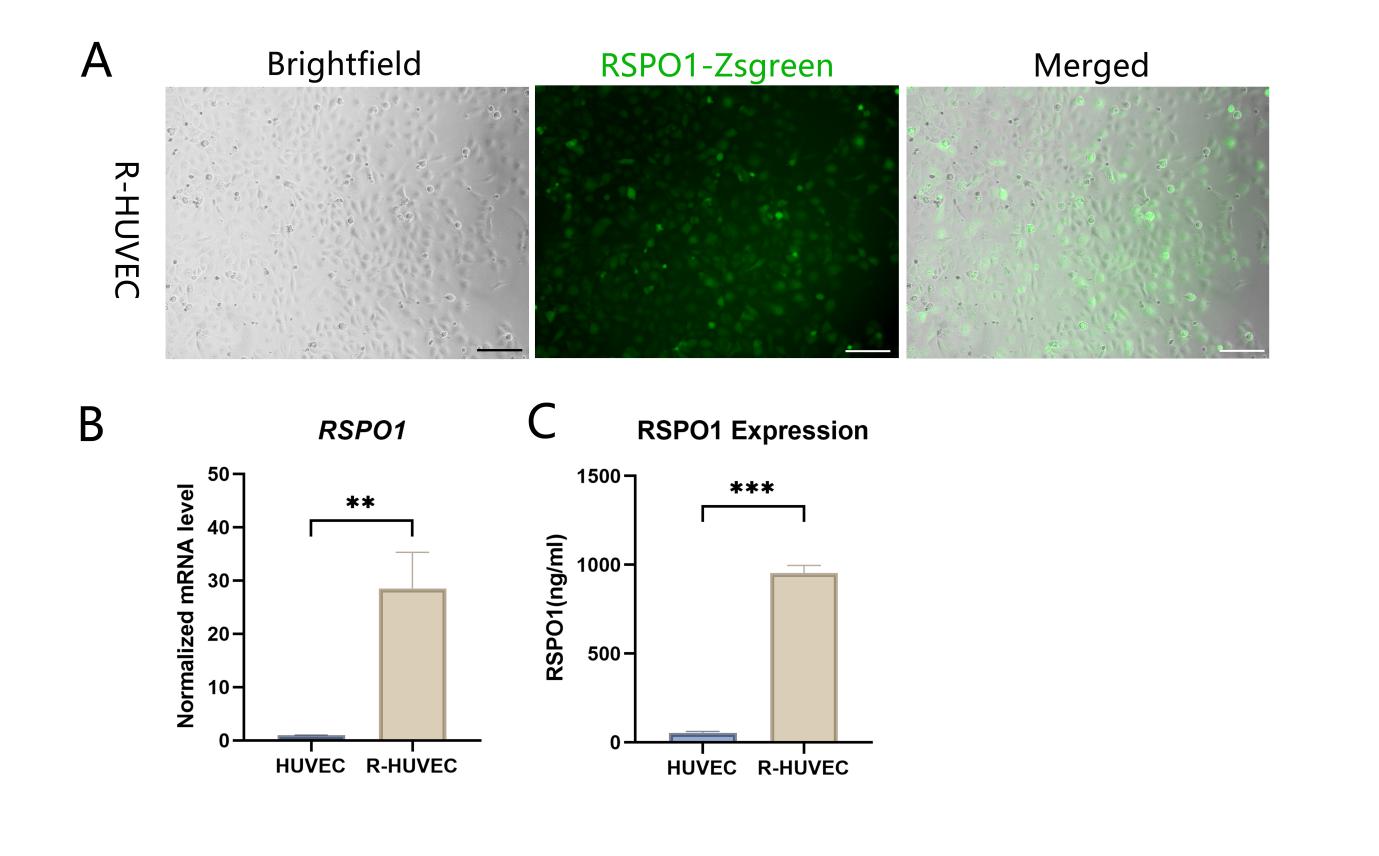


**Figure S1 Establishment of R-HUVEC cells overexpressing RSPO1 protein.**

1. *RSPO1* gene was successfully transfected into HUVEC. Brightfield imaging (left), fluorescence (middle) and merge (right) image of R-HUVEC. Scale bar = 50 μm.
2. Quantifcation of the gene expression by RT-qPCR of RSPO1. Data were normalized to the expression of HUVEC ( ***p*<0.01, n=3).
3. The expression of RSPO1 protein was detected by ELISA (****p*<0.001, n=3).
